# Pecuniary and Non-Pecuniary Incentives to Increase the Rate of Organ Donations from the Living: A Moral Exploration

**DOI:** 10.5041/RMMJ.10050

**Published:** 2011-04-30

**Authors:** Michael Y. Barilan

**Affiliations:** Department of Medical Education, Sackler Faculty of Medicine, Tel Aviv University, Tel Aviv, Israel

**Keywords:** Organ transplantation, live donors, financial incentives/rewards, market in organs, altruistic donation

## Abstract

This paper examines the morality of schemes of payment to live donors/sellers of organs for transplantation. Following empirical and historical evidence, it is argued that consent to sell organs is substantially different from consent to ordinary business transactions and that legalization of exchanges of organs with financial benefits deviates significantly from the scope of liberal toleration and liberal conceptions of human rights. Although altruistic giving is commendable, it is immoral for society to benefit from them without conferring to the donors benefits such as health and nursing insurance for life. Non-alienable and non-fungible benefits of this kind are moral as incentives to organ donation/giving.

## INTRODUCTION

Every day patients die while on a waiting list for kidney transplantation. Many cadaveric organs are not transplanted because of legal constraints or refusal of next of kin. Anybody who is committed to the value of life and human dignity cannot accept this grim situation.

In many places of the world, money is offered to healthy people in exchange of a kidney donation. Similar schemes might be conceived regarding lobes of lung and liver. The probity of pecuniary payments, compensations, or incentives offered to such so-called donors is hotly debated worldwide.

Language is a central player in the debate, as the very vocabulary used predetermines much of the discussion. Therefore, in this paper I use words such as “donors” and” sellers” as synonyms, and instead of the terms “compensation” and “payments” I speak about “pecuniary incentives”, having in mind all sorts of cash or liquid benefits that are offered to people in order to encourage them to give a kidney for the sake of transplantation in a needy patient.

In the forthcoming discussion I will argue that fungible incentives are immoral and should be prohibited even if they are likely to increase the rate of transplantation and of health indexes overall; and that society should offer some non-fungible incentives or rewards to every live donor regardless of the motivation or whether he or she asks for it.

This paper discusses kidneys, because kidney transplantations considerably outnumber other forms of organ transplantation. The discussion, however, is relevant to any form of transplantation that may originate from live donors. Kidney and liver lobes are two notable examples.

## AN OVERVIEW OF DIFFERENT MODES OF ORGAN PROCUREMENT

Of all possible schemes of organ procurement, only one benefits from public consensus – altruistic donations (including presumed consent to donate altruistically) from either the living or the dead. Consensus prevails also with regard to two prohibitions – against non-consensual harvest from live donors and against consensual but harmful removal (e.g. a parent with a single kidney who wishes to donate to a child). Although the ethics of one scheme may depend on the morality of another, the academic literature on organ procurement tends to focus each time on a single scheme. For example, it seems reasonable to assume that the morality of markets in organs from the living may depend on whether non-consensual harvest from the dead is practiced first or whether all potentially eligible cadaveric kidneys have been used. But publications promoting markets in organs ignore this approach (e.g. Cherry^1^).

Currently, there is some literature in favor of non-consensual harvest from the dead; more intense is the debate on markets for organs from the living. The two approaches aim at expanding the availability of organs, but they are not conceptually compatible with each other. Support for non-consensual harvest from the dead is based on utilitarian considerations, whereas libertarians typically respect every personal choice including the choice to sell one’s own kidney at “market price”. The utilitarians are committed to the fair promotion of personal happiness, while the libertarians respect personal choices regarding body and self, regardless of its actual impact on well-being. Utilitarians would support non-consensual harvest from the dead; the libertarians would not.

Opponents to a market in organs contend that commercialization of the human body is considered offensive to human dignity, exploitative of the vulnerable, and an act that by its own nature cannot reflect autonomous choices of people free from formidable constraints (e.g. Delmonico and Scheper-Hughes^2^). Supporters invoke the values of liberty, “self-ownership”, and the saving of human lives. They also believe that at least “well ordered” societies can regulate markets in organs, thus eliminating many issues of justice (e.g. Hippen and Matas^3^).

## PARADOXES AND ABSURDITIES IN THE CURRENT STATE OF THE DEBATE

Some people believe that the issue at stake is respect for personal autonomy. Free and informed persons have the power to use their own selves and bodies in the manner that promotes best their well-being overall (e.g. Sreenivasan^4^). Outlawing markets in organs is actually an offense against interested sellers.

But this argument is flawed. Public and medical support to markets in organs is limited to transplantation medicine only. If people are autonomous to sell parts of their bodies, they should have the option to do so to anybody willing to pay. Even within transplantation medicine, nobody suggests that a healthy person sell a cornea in order to save another from blindness. Although it is possible to set a price on the loss of vision in one eye and on the value of obtaining vision at least in one eye, medical ethics is very strongly committed to the value of no harm. The allegedly innocuous nature of losing one kidney is fundamental to the arguments in favor of harvest from the living. This is precisely the point I will tackle later on in the paper.

Consider the following hypothetical scenario. George has made a decision to sell one kidney and use the money to pay for his university tuition. His neighbor is willing to pay him twenty thousand euro for it. This is a considerable sum of money which George has no other chance of making. But there is a better deal still. George’s friend is willing to pay twenty-five thousand euro for using the kidney in his scientific research. If selling a kidney is a question of personal autonomy, then we expect George to jump on the second deal. But nobody I know endorses transactions of this kind. If George is HIV-positive, his kidneys do not qualify for transplantation. Is George being discriminated against when society does not allow him to sell a kidney for research, even medical research that requires a live HIV-positive kidney? The absurdity of these scenarios illustrates clearly that permission to give an organ for transplantation is neither a power (right) nor interest of the giver. People have no claim on society to help them sell organs, no matter how pressing is their need for money. If destitute, they may deserve charity directly, but not through the marketing of their bodies.

In a similar vein, we do not expect society to build brothels where patients and family members may prostitute themselves in order to support organ transplantation and other expensive and life-saving treatments. Many believe that society should offer basic health care for free; libertarians would tolerate self-chosen prostitution whatever the ultimate goal might be. The notion of state-run trade in sex is unheard of, even if it is directed and limited to saving life. The only reason why many physicians and ethicists promote markets in organs and not markets in sex is that removal of kidneys is done in a surgical theatre, while prostitution is not medicalized; the kidney taken is the one transplanted, while money operates as an agent between the sex and the needed treatment. I do not see why such differences should be relevant to a well ordered society that can regulate markets.

Perhaps, in order to extract ourselves from this speculative quagmire, we had better examine the only scheme of procurement that is at the heart of the consensus – altruistic donation.

Whereas a market in organs is based on *consent* to sell, altruistic donation is based on a *pre-meditated desire* to *help.* Typically, people consent because they *have to;* had they had alternatives, they would not give away body parts. Altruistic donors donate because the *receivers* have no alternative. Put in other words, the “free gift” is immune to duress and unconscionability.^5^ Hence, only with regard to altruistic donations may the value of respect for personal autonomy and human dignity be applied with confidence. Sellers have the power to choose among alternatives; but they are powerless to choose not to face those alternatives in the first place.^6^

Indeed, research on sellers of organs has shown that they come from the poorest strata of society. Almost all of them explain the choice as one made under the pressure of heavy debt or sudden “catastrophic expenditure”, such as a need for expensive health care for a family member; People offering kidneys with self-promotive intentions are a rarity. University tuition or a coveted yacht does not motivate ordinary people to sell kidneys. Only the poor do so, and they do it with redemptive, not promotive, intentions. They seek to cope with a life crisis or to deliver themselves from debt. One may expect these unfortunate people to benefit from the selling in the long run. But they do not. Within a few years, they are burdened with debt once again.^7^,^8^

Precisely here lies the difference between ordinary and rapacious markets. In the ordinary markets for cars and houses, we find people from all walks of society buying and selling. Some are on the way up (they sell in order to get for themselves something better); some on the way down (sell a house in order to pay a debt). Even sellers whose situation is quite distressing retain the chance of buying their goods some day in the future. But the market in organs has only the helpless on the side of supply; they have no promotive intentions and no chances of recuperating their organs in the future. As we have just seen, they even fail to halt their social down-fall.

In the past, defaulting debtors were sent to prison or their children were sold to slavery. But religious, humanistic, and utilitarian considerations stopped such practices. The Hebrew Bible forbade usury and introduced the Jubilee and other laws absolving debts and restoring foreclosed sureties. Christianity and Islam adopted anti-usury laws as well. Private and public charities were created and encouraged in order to provide safety nets for the dispossessed. During the rise of capitalism, the introduction of bankruptcy laws followed the increased emphasis on personal responsibility for commercial commitments. On one hand, society cracked down on promisors and debtors who did not live up to their words, no matter the excuse; on the other hand, without the safety-net of bankruptcy, this new sensibility of responsibility drove many honest but unsuccessful people to unacceptable ruin.^9^

People who desperately need money may be more credit-worthy and may benefit from lower interest rates should they abrogate their right to bankruptcy. But this option is not legal. “Shylock’s deals” are not legal either. As strong incentive to payment as it might be, a person cannot promise to have a leg amputated as punishment for failure to pay debt.

But can he pledge a kidney for transplantation? Loss of leg has a deterrent value only; loss of a kidney might have a price tag in the market as well. A desponded person might find himself choosing between defaulting and a last-chance loan with a kidney as a surety. If people have the right to sell a kidney in order to extract themselves from a financial catastrophe, why not allow them to do it in order to avert their fall? Isn’t the duty of the public to promote any volitional scheme that can save lives? Isn’t it a win-win game?

In my view, the root of the answer is this: Kidneys are not recoverable; money is easy to lose. Put together, a person who cannot hold on to his or her kidney is much less likely to be able to retain cash or any other fungible benefit.

If we go back in time, or move to the poorer areas of the world, it might be true that if the only way to save life is by means of paying the poor for their kidneys, it might even make sense to *prohibit* altruistic donation and cadaveric transplantation so as to divert every available penny in the benefit of the most destitute. The dying will live, and the poor will be a little less miserable, at least temporarily. Those who need the money in order to pay for health care will thus be able to save life as well.

This last absurdity highlights a simple reality – any society that can host transplantation medicine can certainly afford basic protection of the poor in terms of social aid and regulation of the market in credit. Once we acknowledge the full scale of economic power and development that is necessary for transplantation medicine, we may realize that alternative and more responsible schemes for the procurement of organs exist.

An additional consideration is lack of evidence that removal of a kidney is not harmful in the long run to the kind of people who wish to sell – poor day-laborers without healthcare and safe-working environment. Long hours of physical work in the fields of Pakistan and the sweatshops in China might present extraordinary psychological loads on a single kidney. In sum, since good reasons show that sellers are even worse off in psycho-social terms, medicine and society must not endorse and participate in schemes of money-for-kidneys. Doing so is incompatible with the values of medicine and with the accepted range of self-regarding choices that people enjoy in liberal and communitarian societies alike.^10^

## TWO POSSIBLE ALTERNATIVES

### NON-CONSENSUAL HARVEST FROM THE DEAD

If we believe that medicine and society are deeply committed to saving lives, we must not only object to immoral schemes of organ procurement but also search for acceptable ones.

One reason why interest in incentives to live donations is rising is the low rate of cadaveric donations. It is a commonplace tenet that compulsory harvest from the dead might erode public trust in medicine. I am not sure. I am deeply worried about the possibility that society cares to respect the dead, and people’s sentiments regarding “their” dead, more than the health and lives of those still living.

One unexplored scheme is payment to next of kin for consent to donate. This will be expensive, because it will create expectation to be paid for every donation from the dead. But it might still be worthwhile to pay more money and save more lives overall. We know that many objections to donations from the dead are rooted in the wishes and values of the deceased persons and their families. Problems of trust in medicine might also stymie consents to donation. Precisely because payment for consent might work, this would be inappropriate, as society does not try to bribe people against their values and judgment. In this light the use of money to push people to harm their own bodies against their values and judgment seems even more out of place. Even if the harm to the seller is very minor, it is certainly more significant than the harm incurred by harvest from a dead relative.

It is reasonable to increase the price offered when somebody is unwilling to sell a coveted property. But coveted kidneys are not property in waiting for the optimal opportunity for consumption or liquidation. This is why they have no price tag in the first place, and this is why offering money in exchange of kidneys will not make it worthwhile to sell, even though many might consent to sell when prices are high enough (e.g. millions, tens of millions). It is amazing to find ethicists who are confident about the moral duty to set limits on public expenditure on health care but at the same time do not find the expenditure on live organs excessive. They would allow patients to die when treatment for their conditions is too expensive, protecting public funds more than they care to protect desperate people.

Because we do not want society to maim the living and leave the dead intact, it may be argued that no efforts should be made to encourage live donations/sales at all. One possible response might invoke the estimate that even if all eligible cadaveric kidneys are donated, there will still be a shortage in organs.^11^ An additional response might be that should live donors/sellers be given a truly basic good they cannot otherwise receive and which is at the level of the good lost (the kidney), procurement from live people might be a reasonable course of action, especially when the alternative is forceful harvest from the dead.

### NON-PECUNIARY LIFE-LONG AND INALIENABLE BENEFITS AS INCENTIVES TO LIVE DONATIONS

A society that is genuinely motivated by care for basic human values must not promote transplantation by encouraging people to waive their right to bodily integrity. In order for transplantation from the living to be morally valid, organs should be taken from people who desire to do so, and with good reasons. Hence, the incentive to be offered must be substantial, inalienable, and inevitably good for the personal well-being of the donors/sellers.

I believe that non-fungible and lasting basic human goods (hence PGB), such as health insurance and nursing insurance for life, may constitute moral and effective incentives for live donors. As a matter of fact, I will argue that society has the moral duty to confer such goods on altruistic donors as well; that it is immoral to accept organs from well intentioned donors without giving them health care when they need it themselves, whenever society can afford such care.

Kant divided everything that exists into objects that are tradable and consequently have market value, and persons who are unique and irreplaceable and consequently have dignity that is beyond value. One needs not be a Kantian in order to realize that whereas every person may accumulate and may lose money and property, it is only possible to lose body parts, not acquire them. Once given away, they are irreplaceable. It is impossible to undo the violation of the body and the invasion of a person. Hence, nobody wants to give a kidney. People either consent due to dire circumstances or want to help a needy person. In the absence of pressing exigency of either the self or a needy other, nobody will consent to the removal of a healthy kidney.

The typical poor seller cannot retain any future benefit from the money collected, since the money disappears in the form of payment for an old debt or is directed to cover an unexpected life-cycle need. Even sellers who manage to retain the revenue are always at risk of losing it in the future. In terms of security and reversibility, fungible benefits cannot match the loss of a vital organ. However, when a person receives health insurance and similar personal and non-transferable benefits, their values last for the rest of his or her life. Moreover, the transaction redeems the person (and his or her immediate family) from worry about falling into crises of the kind that stimulates sells of organs.

Since every society that supports organ transplantation can also afford PGB to the donors/sellers, abstention from doing so seems immoral. This is especially relevant with regard to the United States and other OECD countries, which can easily afford high-quality health and nursing insurances for donors, whose donations will reduce the financial burden of dialysis care.

Moreover, as much as altruistic giving is commendable, taking without reciprocal giving is not. A helpless needy recipient is not bound to pay back his altruistic donor; but society, which benefits from the donation as well, has a moral duty to care for the donors. People who risk their health by handing over a vital organ certainly deserve health care and nursing care when they need it.

When people seek to give kidneys in exchange for PGB, all other people involved in the process know that the harm incurred is reciprocated by a substantial human good which is lasting and promotive of the human dignity of the persons. The persons giving the organs are less likely to regret it in the future, since the benefit incurred will last for the rest of their lives and is directly related to the very risks involved in losing a kidney. It also contributes directly to saving human life and to amelioration of suffering. Many people will not consider PGB as either incentives or rewards, but as moral duties owed by society to altruistic and non-altruistic givers of organs for transplantation. Perhaps some altruistic donors hesitate because of fears of future medical complications so as to render PGB a removal of disincentives rather than as incentives or rewards. Moreover, once we conceptualize and institute PGBs as moral duties to live organ donors, PGB may not be considered incentives or rewards anymore.

Critics might point out that PGBs might fail because people respond to immediate cash and not to long-term benefits, their ultimate value notwithstanding. Consequently, PGB will not work as efficient incentives, and the supply of kidneys will lag behind that which is expected in so-called free markets for organs. But, in a second thought, this very possibility is one more argument against social participation in markets for organs. If, indeed, people would sell kidneys for ready cash and not for health insurance whose value is much higher, this is a reason not to buy from them, as such business will be clearly harmful, even exploitative.

## SUMMARY

Removal of kidneys from healthy consenting people is a medical procedure, indistinguishable from many other surgeries performed routinely. If the donor acts on genuine altruistic motivations and his or her vital interests are secured, the act is laudable. Reflection on these widely accepted propositions must not blind our eyes to the fact that transactions of kidneys for money offend the values of clinical medicine and are inconsistent with acceptable liberties and protections of the integrity of persons.

Consent in terms of a waiver may suffice in ordinary market transactions, not when the integrity of the body and the dignity of the person are at stake.

Pluralistic democratic society distinguishes between private life and the public sphere. Hence, some liberal societies tolerate voluntary choices to self-alienate interests protected by human rights. Thus, prostitution and bodily mutilation are tolerated. However, the enterprise of organ transplantation is not possible unless society takes an active and central role, where high-tech public medicine (even under private ownership) is the only arena possible. Consequently, society cannot apply the standards of tolerance and negative liberty of the private sphere to organ transplantation. Since only affluent societies can operate organ transplantation routinely, such societies are expected to confer upon organ givers lifelong medical and nursing insurance and possibly additional non-fungible and non-alienable benefits that meet basic human needs.
